# Molecular Mechanism of Antioxidant and Anti-Inflammatory Effects of Omega-3 Fatty Acids in Perilla Seed Oil and Rosmarinic Acid Rich Fraction Extracted from Perilla Seed Meal on TNF-α Induced A549 Lung Adenocarcinoma Cells

**DOI:** 10.3390/molecules26226757

**Published:** 2021-11-09

**Authors:** Payungsak Tantipaiboonwong, Wittaya Chaiwangyen, Maitree Suttajit, Napapan Kangwan, Sirichat Kaowinn, Chakkrit Khanaree, Wanisa Punfa, Komsak Pintha

**Affiliations:** 1 Division of Biochemistry, School of Medical Sciences, University of Phayao, Phayao 56000, Thailand; payungsak.t@gmail.com (P.T.); wittaya.ch@up.ac.th (W.C.); maitree.suttajit@gmail.com (M.S.); 2 Division of Physiology, School of Medical Sciences, University of Phayao, Phayao 56000, Thailand; napapan.kangwan@gmail.com; 3 Department of General Science and Liberal Arts, King Mongkut’s Institute of Technology Ladkrabang Prince of Chumphon Campus, Pathiu, Chumphon 86160, Thailand; sirichat29@gmail.com; 4 School of Traditional and Alternative Medicine, Chiang Rai Rajabhat University, Chiang Rai 57100, Thailand; chakkrit.kh@gmail.com (C.K.); wanisapun@hotmail.com (W.P.)

**Keywords:** *Perilla frutescens*, perilla oil, perilla seed meal, omega-3, rosmarinic acid, inflammation

## Abstract

Industrially, after the removal of oil from perilla seeds (PS) by screw-type compression, the large quantities of residual perilla seed meal (PSM) becomes non-valuable waste. Therefore, to increase the health value and price of PS and PSM, we focused on the biological effects of perilla seed oil (PSO) and rosmarinic acid-rich fraction (RA-RF) extracted from PSM for their role in preventing oxidative stress and inflammation caused by TNF-α exposure in an A549 lung adenocarcinoma culture model. The A549 cells were pretreated with PSO or RA-RF and followed by TNF-α treatment. We found that PSO and RA-RF were not toxic to TNF-α-induced A549 cells. Both extracts significantly decreased the generation of reactive oxygen species (ROS) in this cell line. The mRNA expression levels of IL-1β, IL-6, IL-8, TNF-α, and COX-2 were significantly decreased by the treatment of PSO and RA-RF. The Western blot indicated that the expression of MnSOD, FOXO1, and NF-κB and phosphorylation of JNK were also significantly diminished by PSO and RA-RF treatment. The results demonstrated that PSO and RA-RF act as antioxidants to scavenge TNF-α induced ROS levels, resulting in decreased the expression of MnSOD, FOXO1, NF-κB and JNK signaling pathway in a human lung cell culture exposed to TNF-α.

## 1. Introduction

*Perilla frutescens* L., *Nga-mon* in Thai, is an edible food and medicinal herb grown in parts of certain Asian countries, including Thailand, China, Korea, Japan, India, and Laos. Perilla seeds (PS) have been traditionally utilized as a local medicine to treat mental stress and related symptoms or illnesses such as anxiety, asthma, vomiting, coughing, allergies, intoxication, abdominal pain, and indigestion, whereas natives of northern Thailand usually consume sticky rice mixed with ground PS, called as “Kao-Nuk-Nga” [1,2]. Dietary PS provide valuable nutrients and hydrophilic antioxidants and anti-inflammatory phytochemicals such as phenolic acids and flavonoids, mainly rosmarinic acid (RA), apigenin, luteolin, procyanidins, phytic acid, catechin, caffeic acid, chrysoeriol, ferulic acid, and quercetin as well as essential oils, triterpenes, carotenoids, phytosterols, tocopherols, and policosanols [3,4].

Perilla seed oil (PSO) mostly contains essential α-linolenic acid (ω-3 fatty acid) and linoleic acid (ω-6 fatty acid) as well as phytosterols, tocopherols, squalene, and other fatty acids. [5,6]. Several studies also reported the various health-promoting effects of PSO, including neuroprotective effect, learning and memory, and lowering of blood lipid levels [7,8,9,10,11]. Moreover, PSO reduces inflammation and has antitussive effects [12]. Perilla seeds present antioxidant, anti-allergic, anti-asthmatic, anticancer, antidepressant, antimicrobial, antitumor, and antiviral activity [3,13]. Perilla seed meal (PSM), a by-product of PSO extraction process, is discarded as a waste product or used as a protein source in animal feed as a low-cost material [14,15,16]. The PSM should be analyzed and reused because it is rich in numerous bioactive components such as phenolic compounds and others [17,18,19,20]. Because their molecular mechanisms remain unknown, it is worth studying these active pharmaceutical ingredients in PSO and PSM.

Pro-inflammatory cytokines, such as interleukin (IL)-6, IL-1β, and tumor necrosis factor-α (TNF-α), promote cancer progression. TNF-α, a traditional pro-inflammatory cytokine, plays an important role in promoting lung inflammation [21,22]. It has been reported that TNF-α upregulates the expression of c-Jun N-terminal kinases (JNKs), forkhead box class O1 (FOXO1), and manganese-containing superoxide dismutase (MnSOD). An increase in the production of reactive oxygen species (ROS) occurs through many inflammatory signaling pathways such as JNK signaling pathway and upregulation of downstream targets including the transcription factors NF-κB and FOXO1 [23]. In cancer cells, FOXO1 plays an important role in cellular stress response and antioxidant defense mechanisms through upregulating MnSOD [24]. Several studies have indicated that MnSOD overexpression is also associated with the progression of lung cancer and other [25,26,27]. However, the molecular mechanism of PSO and RA-RF on the inhibitory effects in inflammation associated molecules such as JNK, FOXO1, MnSOD and NF-κB have not been fully understood.

Lung cancer has a high prevalence in the North and Northeast of Thailand [28,29]. Several studies have provided evidence to support that inflammation can initiate or promote the development of lung cancer [30,31]. TNF-α, as a biomarker in lung cancer patients [21,32], has an established involvement in inflammation and also plays a key role in inflammation-induced cancer in vitro and in vivo [33,34]. In our previous study, we found that RA, which is predominant in PSM, contributes to its anti-inflammatory and antioxidant activity and health-promoting effects. Owing to these properties, PSO and PSM have recently gained popularity for use in pharmaceutical applications [35,36]. However, the anti-inflammatory effects of PSO and RA-RF on TNF-α-induced A549 human lung epithelial cells and the underlying mechanism have not been investigated. Therefore, the present study aims to investigate the antioxidant activity of PSO and RA-RF from PSM on TNF-α-induced A549 cells. Furthermore, we hypothesized that PSO and RA-RF could reduce ROS levels and inflammation by examining intracellular ROS levels and expression levels of IL-1β, IL-6, IL-8, TNF-α, COX-2 inducible enzymes, FOXO1, and MnSOD. The underlying mechanism will be investigated by Western blot analysis to determine the protein expression of JNK, FOXO1, MnSOD, and NF-κB signaling molecules.

## 2. Results

### 2.1. Preparation and Analysis of Fatty Acids in PSO

Perilla seed oil (PSO) was obtained using a screw-type compressor; its yield was 28.00 ± 0.45% (*v/w*). Further analysis of PSO showed 59.89 ± 1.34% of α-linolenic acid (*ω*-3), 17.70 ± 0.07% of linoleic acid (*ω*-6), 11.81 ± 0.58% of oleic acid (*ω*-9), and other fatty acids including palmitic acid, stearic acid, and oleic acid. Our previous study found that α-linolenic acid, an essential omega-3 fatty acid, was the most abundant polyunsaturated fatty acid in PSO [37].

### 2.2. Partial Purification and Polyphenolic Quantification of RA-RF

Perilla seed meal (PSM) was extracted with 70% ethanol; the yield of extraction was 5.63 ± 0.27%. Partial purification of rosmarinic acid (RA) from PSM was performed using the solvent-partitioning method. Subsequently, four fractions in different solvents-Hexane (Hex), Dichloromethane (DCM), Ethyl acetate (EA), and water were obtained.

The extraction yield, total phenolic content (TPC), total flavonoid content (TFC), and RA content are shown in Table 1. The TPC in the ethanolic extract was 84.81 ± 0.85 mg GAE/g extract. The TPC in the HEX, DCM, EA, and water fractions was 4.74 ± 0.80, 198.36 ± 1.67, 248.00 ± 5.14, and 27.31 ± 0.11 mg GAE/g extract, respectively. The TFC in the ethanolic extract was 68.33 ± 1.14 mg CE/g extract. The TFC in the HEX, DCM, EA, and water fractions was 12.17 ± 1.62, 88.84 ± 4.88, 165.21 ± 2.61, and 23.56 ± 0.87 mg CE/g extract, respectively.

**Figure 1:**
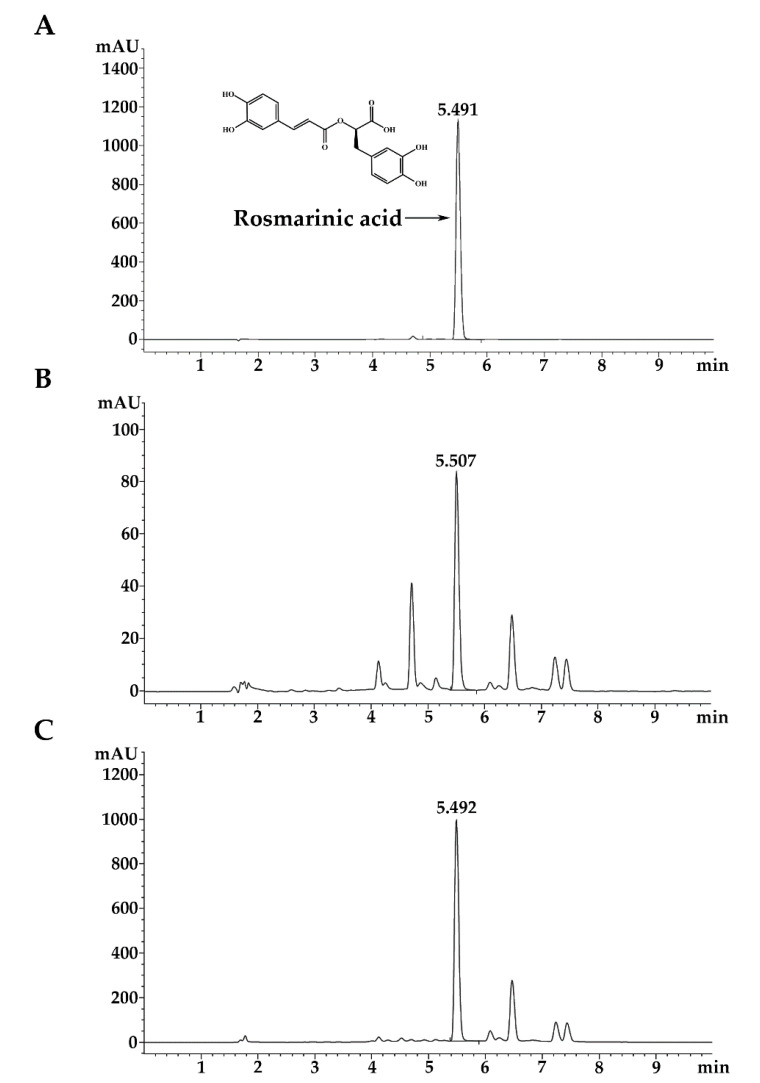
Ultra-high-pressure liquid chromatogram of PSM extract fractions: RA standard (**A**), EtOH (**B**) and EA (**C**).

The RA standard showed the major peak at retention time 5.491 min (Figure 1A). The RA content in 70% ethanolic extract was 71.72 ± 0.72 mg/g extract (Figure 1B). The RA content in HEX, DCM, EA, and water fractions was 54.56 ± 1.63, 29.42 ± 0.20, 647.68 ± 53.08, and 37.66 ± 0.19 mg/g extract, respectively. As indicated in Table 1 and Figure 1C, it can be concluded that the EA fraction contained the highest quantities of TPC, TFC, and RA. This fraction is named as rosmarinic acid-rich fraction (RA-RF) for further characterization of its biological activity.

### 2.3. Effect of PSO and RA-RF on Cell Viability and Morphology

**Figure 2:**
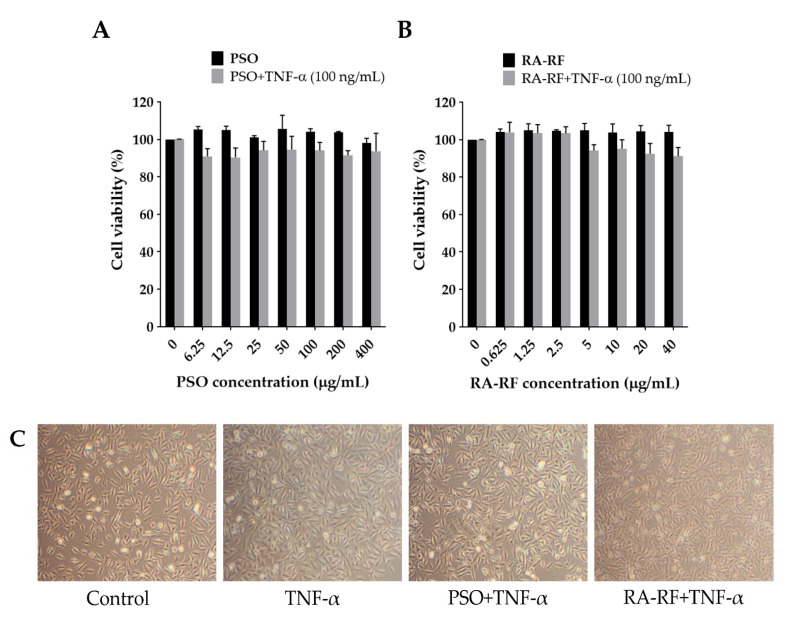
Effect of PSO and RA-RF on A549 cell viability. The cells were pre-incubated with PSO (**A**) or RA-RF (**B**) for 2 h and then treated with or without 100 ng/mL of TNF-α for 24 h. All assays were performed in triplicate; the data are presented as mean ± standard deviation. Morphology of A549 cells treated with 0.5% DMSO, 100 ng/mL of TNF-α, PSO (400 µg/mL) + TNF-α (100 ng/mL), and RA-RF (40 µg/mL) + TNF-α (100 ng/mL) (**C**) observed under inverted phase contrast microscopy (100× total magnification).

The cytotoxicity of PSO and RA-RF was determined in A549 cells using the MTT assay. The A549 cells were seeded into 96-well plates at 5.5 × 10^3^ cells/well and incubated overnight. Next, the cells were pretreated with PSO (0–400 µg/mL) and RA-RF (0–40 µg/mL) for 2 h and co-treated with or without TNF-α (100 ng/mL) for 24 h. As shown in Figure 2A,B, PSO and RA-RF had no effect on A549 cell viability. The inhibitory concentrations at 20% (IC_20_) and 50% (IC_50_) of A549 cell survival were higher than 400 µg/mL for PSO and 40 µg/mL for RA-RF. To determine the morphological alterations, cells were treated with TNF-α, PSO, RA-RF or in combinations at high concentrations and observed over 24 h under a microscope. Neither TNF-α, PSO, RA-RF, nor co-treatment could change the morphology of A549 cells (Figure 2C).

### 2.4. Effect of PSO and RA-RF on TNF-α-Induced A549 Cells Apoptosis

**Figure 3:**
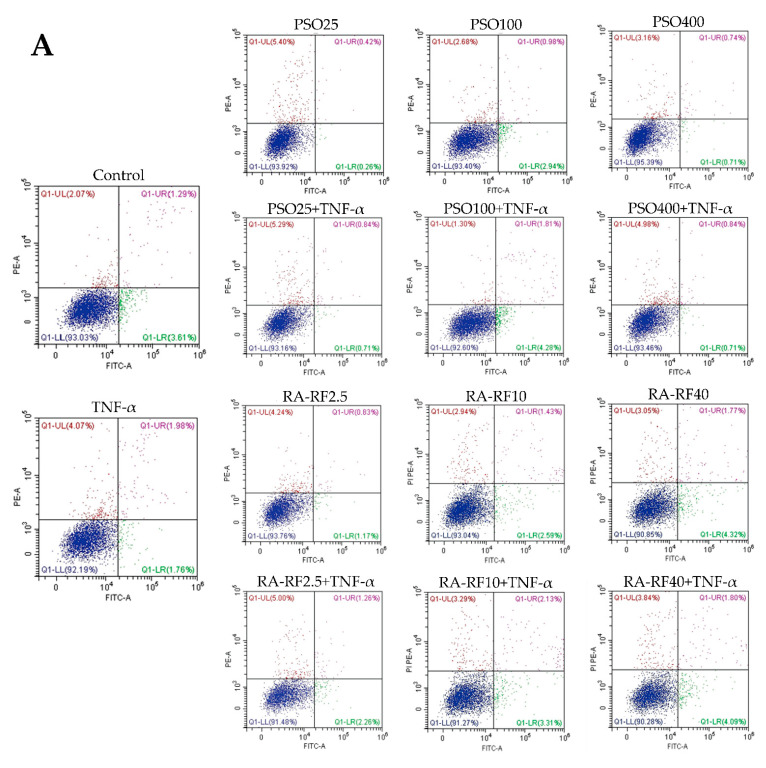

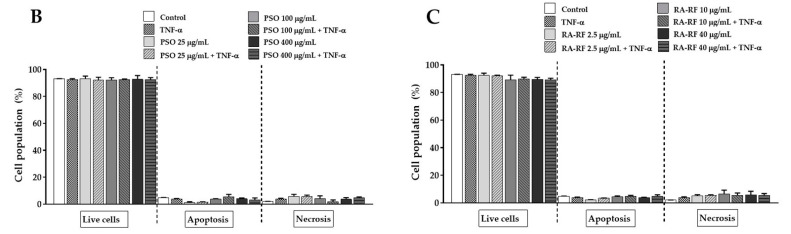
Cell apoptosis and necrosis of A549 cells upon the treatment of PSO (25, 100, and 400 μg/mL) or RA-RF (2.5, 10 and 40 μg/mL), followed by treatment with or without 100 ng/mL TNF-α. Cells were then analyzed using flow cytometry. Dot plots show annexin V-FITC+/PI- as early apoptotic cells, annexin V-FITC+/PI+ as late apoptotic cells, and annexin V-FITC-/PI+ as necrotic cells (**A**). Live, apoptotic, and necrotic cells were presented as a percentage of cell population in the bar graphs of PSO (**B**) and RA-RF (**C**). The data were reported as a mean ± SD of three independent experiments carried out in triplicate.

The apoptosis assay was used to confirm the A549 cell viability upon the combination treatment between PSO (25, 100 and 400 µg/mL) or RA-RF (2.5, 10 and 40 µg/mL) with or without 100 ng/mL TNF-α. As shown in Figure 3A–C, the treatment of PSO and RA-RF alone or co-treatment with TNF-α had no effect on cells apoptosis and necrosis in A549 cells when compared to the control.

### 2.5. Effect of PSO and RA-RF on Intracellular ROS Production

**Figure 4:**
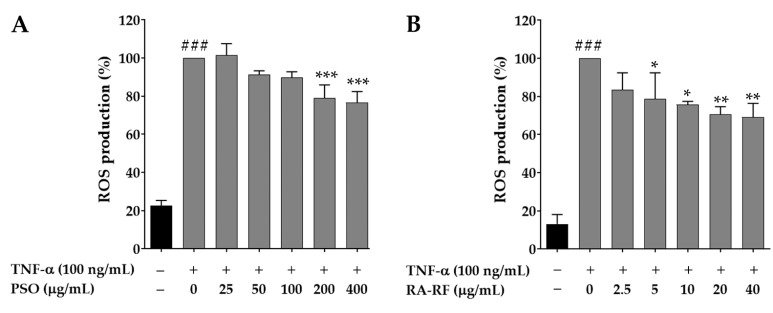
Treatment with PSO (**A**) and RA-RF (**B**) reduced ROS levels in TNF-α-treated A549 cells. All assays were performed in triplicate; data are presented as mean ± standard deviation (^###^ *p* < 0.001 vs. the control group; * *p* < 0.05, ** *p* < 0.01, *** *p* < 0.001 vs. (+) TNF-α induced group).

The intracellular ROS production was measured by the DCHF assay that has been widely used in oxidative stress determination. To monitor the intracellular ROS level in A549 cells, the cells were pre-treated with PSO or RA-RF, and then TNF-α (100 ng/mL) was added. The co-incubation with DCFH-DA was performed for 30 min. As seen in Figure 4A,B, TNF-α-treated cells showed significantly higher intracellular ROS levels than non-TNF-α-treated cells (*p* < 0.001). Treatment with PSO (200 and 400 µg/mL) and RA-RF (5–40 µg/mL) significantly reduced intracellular ROS production in the TNF-α-treated cells.

**Figure 5:**
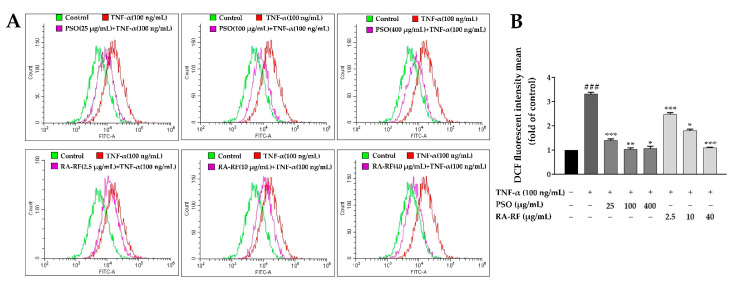
Reactive oxygen species (ROS) generation by flow cytometry. ROS generation level is exhibited as mean ± SD of fluorescence intensity in folds compared to that of the control (without TNF-α) from three independent experiments by using fluorescence probes: DCFH-DA (**A**). A Bar graph depicted the DCF fluorescence intensity of PSO (25, 100 and 400 µg/mL) or RA-RF (2.5, 10 and 40 µg/mL) treated sets, followed by treatment with or without 100 ng/mL TNF-α in A549 cells (**B**). All assays were performed in triplicate; data are presented as mean ± standard deviation (^###^ *p* <0.001 vs. the control group; * *p* < 0.05, ** *p* < 0.01, *** *p* < 0.001 vs. (+) TNF-α induced group).

Consequently, to confirm whether or not PSO or RA-RF reduced ROS production in A549 cells, intracellular ROS were determined by using Flow Cytometry. The results were similar to those of the DCHF assay (Figure 5A,B). Thus, exposure to PSO and RA-RF effectively prevents the production of ROS that cause cellular oxidative damage.

### 2.6. Downregulation of IL-1β, IL-6, IL-8, TNF-α, and COX-2 mRNA Expression in A549 Cells Treated with PSO and RA-RF

**Figure 6:**
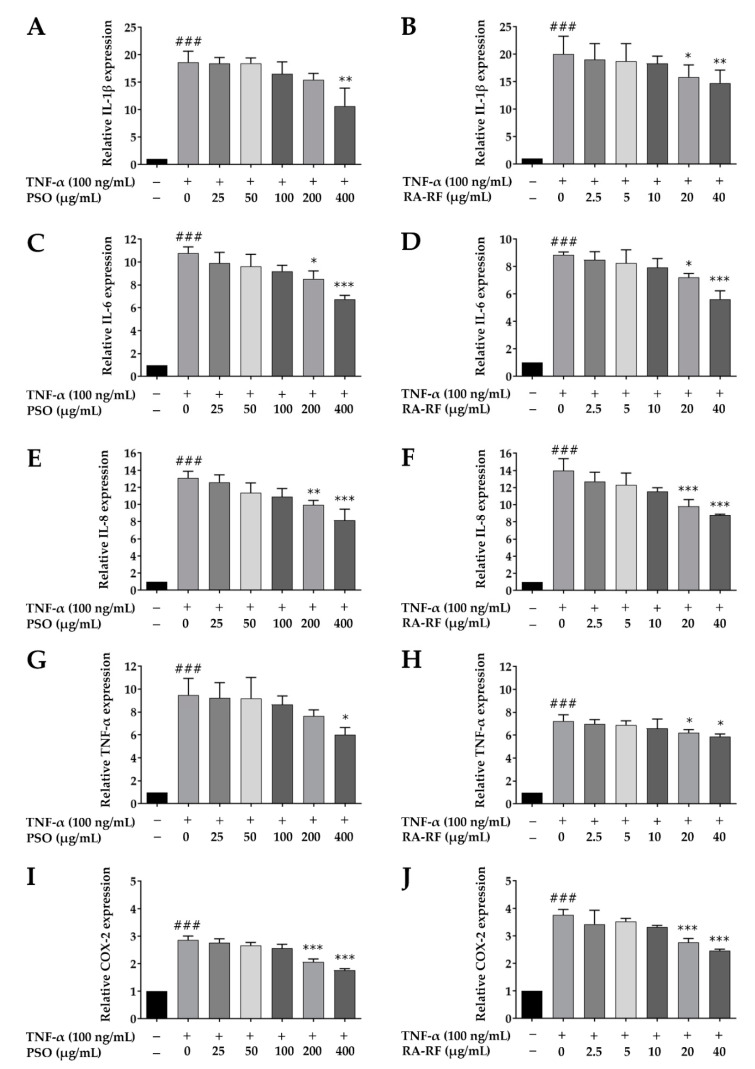
Treatment with PSO and RA-RF downregulated the mRNA expression of IL-1β (**A**,**B**), IL-6 (**C**,**D**), IL-8 (**E**,**F**), TNF-α (**G**,**H**), and COX-2 (**I**,**J**). All assays were performed in triplicate; data are presented as mean ± standard deviation (^###^ *p* < 0.001 vs. the control group; * *p* < 0.05, ** *p* < 0.01, *** *p* < 0.001 vs. (+) TNF-α induced group).

To investigate the protective effects of PSO and RA-RF on the mRNA expression levels of inflammatory mediators, A549 cells were pre-incubated with PSO (25–400 µg/mL) and RA-RF (2.5–40 µg/mL) for 2 h. To induce inflammation, 100 ng/mL of TNF-α was added, and the cells were incubated for another 24 h. The mRNA expression levels of IL-1β, IL-6, IL-8, TNF-α, and COX-2 were then determined using qRT-PCR. As shown in Figure 6A–J, the TNF-α-treated A549 cells significantly increased the expression of IL-1β, IL-6, IL-8, TNF-α, and COX-2 in comparison with the control (*p* < 0.001). Treatment with PSO (400 µg/mL) and RA-RF (20 and 40 µg/mL) significantly inhibited the TNF-α-induced mRNA expression of IL-1β, IL-6, IL-8, TNF-α, and COX-2. Thus, PSO and RA-RF reduce the mRNA expression of inflammatory mediators and subsequently prevent the induction of inflammation.

### 2.7. Suppression Effect of PSO and RA-RF on JNK Phosphorylation

**Figure 7:**
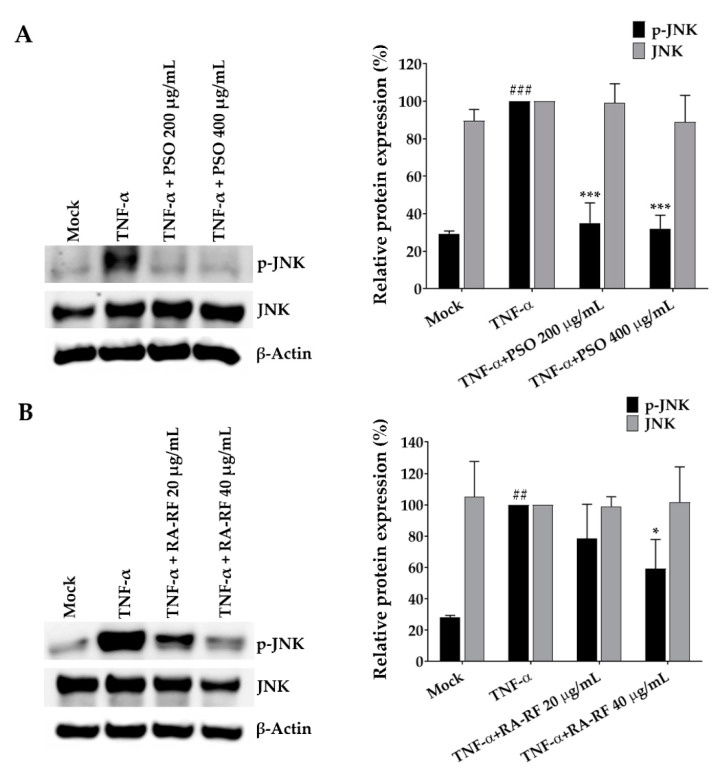
Suppression effect of PSO (**A**) and RA-RF (**B**) on JNK phosphorylation. A549 cells were pretreated with PSO and RA-RF for 2 h and further incubated with tumor necrosis factor-α (TNF-α) for 30 min. Whole-cell lysates were analyzed by Western blotting with anti-p-JNK and anti-JNK antibodies. Actin was used as an internal control. All assays were performed in triplicate; data are presented as mean ± standard deviation (^##^ *p* < 0.01, ^###^ *p* <0.001 vs. the control group; * *p* < 0.05 *** *p* < 0.001 vs. (+) TNF-α induced group).

Several studies have demonstrated that TNF-α induces phosphorylation of JNK, the JNK signal transduction pathway is associated with antioxidation and anti-inflammation, is usually upregulated in cancer cells [33]. The present study further examined the mechanisms underlying the effect of PSO and RA-RF using the Western blot assay. As shown in Figure 7A,B, TNF-α strongly phosphorylated JNK (*p* < 0.001), whereas PSO (200 and 400 µg/mL) and RA-RF (40 µg/mL) markedly decreased the TNF-α-induced JNK phosphorylation. These results indicate that PSO and RA-RF inhibit JNK phosphorylation, which in turn, leads to a decrease of pro-inflammatory cytokine production.

### 2.8. Expression of NF-κB, FOXO1 and MnSOD in A549 Cells

**Figure 8:**
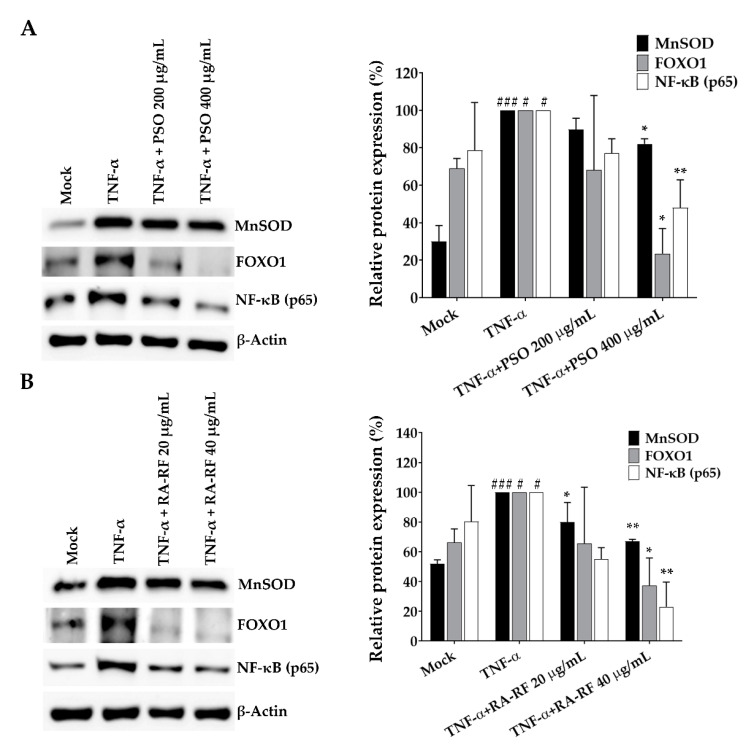
Treatment with PSO (**A**) and RA-RF (**B**) reduced the expression of NF-κB, FOXO1, and MnSOD proteins. A549 cells were pretreated with PSO and RA-RF for 2 h before incubation with TNF-α for 24 h. The cell lysates were analyzed by Western blotting with anti-NF-κB, anti-FOXO1, and anti-MnSOD antibodies. All assays were performed in triplicate; data are presented as mean ± standard deviation (^#^ *p* < 0.05, ^###^ *p* < 0.001 vs. the control group; * *p* < 0.05, ** *p* < 0.01, vs. (+) TNF-α induced group).

In addition to demonstrating a reduction in ROS production and expression of inflammatory cytokines by PSO and RA-RF, we clarified their underlying mechanism related to NF-κB, a signaling molecule involved in the inflammatory and immune [38,39]. This study hypothesized that PSO and RA-RF play a crucial role in the diminished inflammatory response through the inhibition of NF-κB. The TNF-α-treated cells showed significantly higher levels of NF-κB than untreated cells, and PSO and RA-RF treatments markedly reduced the expression level of NF-κB in TNF-α-stimulated A549 cells (Figure 8A,B). This finding indicates that both PSO and RA-RF could exert their inhibitory effect on the TNF-α-induced the up-regulation of IL-1β, IL-6, IL-8, TNF-α, and COX-2 mRNA through the reduction of NF-κB protein expression.

Several studies have demonstrated that FOXO activation plays an important role in the expression of genes associated with stress resistance and MnSOD which is a detoxifying enzyme in the cell [40]. Therefore, we further examined the effect of TNF-α on FOXO1 and MnSOD expression by Western blot. FOXO1 and MnSOD protein levels significantly increased in response to TNF-α whereas the higher dose of PSO and RA-RF distinctly inhibited FOXO1 and MnSOD expression in treated cells. The findings propose that the reduction in protein expression was due to the decreasing ROS levels.

## 3. Discussion

Among the fatty acids contained in PSO, ω-3 fatty acids were present in the highest quantity, followed by ω-6 and ω-9 fatty acids. The ratio of ω-3 fatty acids and ω-6 fatty acids was approximately 3.34:1, which is beneficial for health. Diets in industrialized Western countries usually have excessive quantities of ω-6 fatty acids and are deficient in ω-3 fatty acids [41,42]. Extremely high ω-6:ω-3 ratios ranging up to 50:1 [43] in daily diet markedly increases the risk of many non-communicable diseases, including obesity, cardiovascular disease, cancer, and inflammatory and autoimmune diseases, whereas high ω-3:ω-6 ratios in non-Western diets suppress the risk [44,45,46]. As previously reported by our group, flavonoids and phenolic acids, mainly RA, were predominant in PSO and PSM. These hydrophilic secondary metabolites are well known for antioxidant, anti-inflammatory, and antimutagenic activity [35]. As shown in Table 1, PSM, which is the by-product of PSO, was fractionated by organic solvents. The results showed that the EA fraction contained higher quantities of phenolics (~2.92-fold), flavonoids (~2.42-fold), and RA (≈9.03-fold) than the starting ethanolic extract. This finding is consistent with previous findings that RA was chiefly found in the EA fraction [47,48,49,50].

Reactive oxygen species (ROS) are free radicals in biological systems that exhibit the progression of inflammatory disorders. Many studies have demonstrated that ROS act as a signaling molecule distribute to the gain of various pro-inflammatory cytokines (e.g., IL-1β, IL-6, and TNF-α) [51]. The challenge in discovery of dietary compounds as novel therapeutic approaches is to scavenge level of cellular ROS production in inflammatory diseases [52]. The present study investigated whether PSO and RA-RF have the ability to suppress the increased generation of ROS in TNF-α-induced A549 cells. Our data (Figure 4 and 5) revealed a significant reduction in intracellular ROS levels in A549 cells treated with PSO and RA-RF. These findings are consistent with those reported previously. It was found that cold-pressed PSO mainly consisting of ω-3 fatty acid reduced the ROS production induced by ultraviolet radiation in normal human dermal fibroblasts [53], and the EA fraction from PSM, which contained high quantities of RA, inhibited ROS production in RANKL-induced RAW 264.7 cells [54]. Our results suggest that both PSO and RA-RF have antioxidant ability and equally inhibit ROS generation.

Oxidative stress causes inflammation by triggering the inflammatory response. It produces excessive free radicals that lead to further oxidative damage of cellular components and inducing chronic inflammation [55]. Overproduction of pro-inflammatory cytokines such as IL-1β, IL-6, IL-8 and TNF-α is associated with various inflammatory diseases [56]. In our study (Figure 6), both PSO and RA-RF, at non-toxic concentrations, significantly decreased IL-1β, IL-6, IL-8, TNF-α, and COX-2 mRNA expression in TNF-α-induced A549 cells. The results also supported previous findings that suggested that dietary PSO significantly reduced the production of pro-inflammatory cytokines (TNF-α, IL-1β, and IL-6) in a mouse model [57]. Moreover, PSO clinically decreased TNF-α expression in hyperlipidemic patients [11]. Consistent with the anti-inflammatory activity of RA-RF from PSM reduced the expression of COX-2 in RAW264.7 cells activated by LPS and IFN-γ [54]. Moreover, RA-enriched fraction from perilla leaves suppressed the mRNA expression of IL-1β, IL-6, TNF-α, and COX-2 in an animal model [58]. Our results indicate that PSO mainly containing ω-3 fatty acids and PSM extract rich in RA alleviate A549 cell inflammation by downregulating the pro-inflammatory cytokines.

The JNK family are a group of kinases that bind and phosphorylate c-Jun. High JNK activity has been associated with pro-inflammatory cytokine upregulation observed in different cancer cell lines [59]. Phosphorylation of JNK is induced by an increase in the levels of cellular ROS, which promote the production of pro-inflammatory cytokines in response to inflammation [59,60]. Therefore, we hypothesized that PSO and RA-RF, which exert an antioxidant activity against ROS production, may be able to reduce the TNF-α-induced phosphorylation of JNK. Our results showed that TNF-α significantly increased JNK phosphorylation, whereas both PSO and RA-RF suppressed JNK phosphorylation in A549 cells (Figure 7). A previous study reported that ω-3 fatty acid-enriched PSO suppressed the JNK phosphorylation induced by high-fat diet in mice [61]. In addition, RA was reported to decrease the p-JNK/JNK protein expression in a mouse airway inflammation model [62] These findings suggest that PSO and RA-RF exert an inhibitory effect on the JNK signaling cascade and inflammation, which are triggered under elevated oxidative stress in TNF-α-activated cells.

Moreover, the increase in cellular ROS production also triggered the JNK signaling cascade activated by oxidative stress, resulting in FOXO and MnSOD overexpression [40]. Furthermore, we observed that PSO and RA-RF reduced FOXO1 and MnSOD expression in TNF-α-treated A549 cells. Several studies have reported that FOXO transcription factors are expressed in various cell types such as lung, breast, and prostate cells, leading to proliferation of tumor cells especially targeting FOXO1 [63]. Cellular functions such as inflammation are regulated by FOXO1 transcription factors, which leading to overexpression of IL-1β, IL-2 and IL-6 [64,65]. FOXO1 is strongly activated by TNF-α both in vitro and in vivo [64,66]. Remarkably, TNF-α has also been shown to increase ROS production and MnSOD expression in cells, and FOXOs induce MnSOD expression [40].

MnSOD is a powerful antioxidant enzyme that suppresses ROS production [67]. TNF-α could upregulate MnSOD expression in A549 lung adenocarcinoma cells. [68]. Based on our finding that PSO and RA-RF inhibit TNF-α induced ROS production, we hypothesized that FOXO1 and MnSOD expression are adaptive responses to ROS levels. As expected, FOXO1 and MnSOD expression significantly increased upon TNF-α stimulation (Figure 8), whereas PSO and RA-RF treated A549 cells had lower FOXO1 and MnSOD expression. A previous study showed that docosahexaenoic acid (DHA), an ω-3 fatty acid extracted from algae, significantly lowered FOXO1 and FOXO3 expression in the liver and adipose tissue of weaned piglets [69]. Remarkably, commercial ω-3 fatty acids EPA and DHA downregulated the gene expression of antioxidant enzymes including MnSOD and CuZnSOD in doxorubicin-treated A549 cells [70]. However, the cellular effect of RA on FOXO1/MnSOD regulation has been rarely studied and reported.

NF-κB is a transcription factor that is found to be upregulated during inflammation in cell culture and animal models. Several strategies in the treatment of inflammatory diseases include attacking the target of the NF-κB signaling pathway [71]. NF-κB activation leads to increased levels of certain pro-inflammatory cytokines, including IL-1β, IL-6, TNF-α, and COX-2 [38]. Our results clearly illustrated that TNF-α-induced expression of NF-κB protein was significantly decreased by PSO and RA-RF. The findings in our study are in accordance with the report that in mice, PSO rich in alpha-linolenic acid also inhibits COX-2 and iNOS expression by inhibiting the protein expression of NF-κB (p65) [72]. Moreover, it was found that PSO reduces the expression of IL-1β, IL-6, TNF-α, and COX-2 mRNA by inhibiting p65 phosphorylation in colonic inflammation induced by a high-fat diet in mice [73]. Notably, RA alone was shown to inhibit inflammation in H22 tumor-bearing mice by reducing the IL-1β, IL-6, and TNF-α levels and p65 and p-p65 expression [74]. In summary, PSO which is rich in ω-3 fatty acids and PSM extract which mainly contains RA, are able to regulate the expression of inflammatory cytokines via suppression of NF-κB protein expression.

The composition of PSO (ω-3 fatty acids) and RA-RF (rosmarinic acid) is completely different. However, the mechanism for anti-inflammatory effects of PSO and RA-RF in A549 cells is similar. The previous studies demonstrate a role played by an increased tissue status of ω-3 fatty acids and decreased ω-6/ω-3 ratio in modulation of cytokine production, including NF-κB, TNF-α, IL-1β and IL-6. Since NF-κB seems to be the core factor mediating all the effects/interplays, inhibition of NF-κB may be the key target for the ω-3 PUFA’s effects on cytokine production and inflammation. For the mechanism for anti-inflammatory effects RA-RF that contain high content of rosmarinic acid, a previous results showed that rosmarinic acid significantly inhibited the production of IL-6, IL-10, and IL-1β in LPS-treated RAW 264.7 cells by suppression of NF-κB activation [75,76,77,78].

## 4. Materials and Methods

### 4.1. Preparation of PSO and RA-RF

Perilla seeds (PS) were collected from Phayao, Thailand. The voucher specimen (Code: QBG-93756) of the plant was provided by W. Chaiwangyen and has been preserved at the Queen Sirikit Botanic Garden Herbarium, Chiang Mai, Thailand. Perilla seed oil (PSO) obtained from PS using a screw-type compressor (Chiang Mai, Thailand) at room temperature, was centrifuged at 4500 rpm for 10 min for removing small particles. Clear yellow oil was collected and stored at 0–4 °C. Fatty acid composition was analyzed by gas chromatographic method [79]. After oil removal by compression, dry powder of seed residue was extracted with 70% ethanol and shaken for 12 h. After ethanol evaporation and lyophilization, the crude extract was redissolved in hexane-water mixture (1:1) and subsequently purified by solvent extraction with hexane (Hex), dichloromethane (DCM), and ethyl acetate (EA), respectively [58]. The quantity of rosmarinic acid (RA) in all fractions was analyzed by ultra-high-pressure liquid chromatography (UHPLC). The fraction with the highest RA content (RA-RF) was used in all experiments.

### 4.2. Determination of Total Phenolic Content

The assay of Folin-Ciocalteu was used for determination of TPC [80]. In brief, the sample was mixed with 10% Folin reagent and 7.5% sodium carbonate and kept at room temperature for 15 min. The absorbance of this solution was measured at 765 nm using gallic acid as a phenolic standard. Total phenolic content was presented as gallic acid equivalents in milligram per gram of extract (mg GAE/g extract).

### 4.3. Determination of Total Flavonoid Content

Total flavonoid content (TFC) was determined by aluminum colorimetric method [80]. The sample was mixed with 5% NaNO_2_ for 5 min. Then 10% AlCl_3_ and 1 M NaOH were added to the mixture, and it was kept at room temperature for 10 min. The absorbance of the mixture was analyzed at 510 nm using catechin as a flavonoid standard. Total flavonoid content was presented as catechin equivalents in milligram per gram of extract (mg CE/g extract).

### 4.4. Determination of RA Content

To determine the RA content [80], the sample fractions from PSM were filtered and loaded on the UHPLC system (Agilent Technologies, Inc., Santa Clara, CA, USA) containing a C18 column (150 mm × 4.6 mm × 5 µm). The UHPLC chromatogram of RA was determined and compared with that of the RA standard. The mobile phase contained a mixture of 0.1% trifluoroacetic acid and acetonitrile. The flow rate was set at 1.0 mL/min and monitor of RA was measured at 280 nm.

### 4.5. Cell Culture

The A549 cell line derived from human lung epithelial cell carcinoma were obtained from the ATCC (Manassas, VA, USA) and cultured in DMEM at 37 °C in a humidified 5% CO_2_ atmosphere. The medium was supplemented with 10% fetal bovine serum and antibiotics (1% penicillin and 1% streptomycin).

### 4.6. Reagents and Antibodies

Antibodies directed against MnSOD, FOXO1 and β-actin were purchased from Santa Cruz Biotechnology (Santa Cruz, CA, USA). Antibodies directed against total and phosphorylated c-Jun N-terminal kinase (JNK), and NF-κB were obtained from Cell Signaling Biotechnology (Danvers, MA, USA). TNF-α was purchased from R & D Systems (Minneapolis, MN, USA).

### 4.7. Cell Viability Assay and Morphological Assessment

The cell viability was examined using MTT assay with some modification [35]. The A549 cells were seeded into 96-well plates at 5.5 × 10^3^ cells/well and incubated overnight. Next, the cells were pretreated with PSO (0–400 µg/mL) and RA-RF (0–40 µg/mL) for 2 h and co-treated with or without TNF-α (100 ng/mL) for 24 h. MTT dye was added to each well; the plates were incubated for 4 h, and then the culture medium was removed. Then, formazan was dissolved in dimethyl sulfoxide, and absorbance at 570 and 630 nm was measured using a spectrophotometer. In each experiment, the RA-RF sample was examined in triplicate. The morphology of A549 cells treated with high doses of PSO (400 µg/mL) or RA-RF (40 µg/mL) and co-treated with or without TNF-α (100 ng/mL) was observed under a bright field using an inverted phase contrast microscopy (Olympus, Tokyo, Japan) at ×100 magnification.

### 4.8. Annexin V-FITC/PI Staining for Apoptosis Quantification Using Flow Cytometry

After 2 h of pretreatment with varying doses of PSO and RA-RF, followed by 24 h of treatment with or without 100 ng/mL TNF-α. Annexin V-FITC/PI Staining was performed according to the procedures of Khaw-on et al. [81]. Briefly, A549 cells were suspended and stained with annexin V-FITC and PI using the Annexin-V-FLUOS Staining Kit (Merck KGaA, Darmstadt, Germany) for 20 min and then analyzed using the DxFLEX Flow Cytometer (Beckman Coulter, Indianapolis, IN, USA). CytExpert for DxFLEX was used to examine the PI-positive population for necrotic cells (Beckman Coulter, Indianapolis, IN, USA).

### 4.9. Effect of PSO and RA-RF on Intracellular ROS Production

Intracellular ROS inhibition by PSO and RA-RF was examined. The A549 cells were seeded into 96-well plates and incubated at 37 °C and 5% CO_2_ for 16 h and then washed twice with phosphate-buffered saline (PBS; pH 7.4) Cells were incubated with the fluorescent dye 2′,7′-dichlorodihydrofluorescein diacetate (DCFH-DA; 20 µM) at 37 °C for 2 h. The excess DCFH-DA was removed by washing twice with PBS. The cells were co-treated with PSO, RA-RF, and TNF-α at proper range of concentrations for another 30 min at 37 °C. The fluorescence intensity of oxidized dichlorofluorescein was measured at excitation and emission wavelengths of 485 nm and 530 nm, respectively [35]. Then, the samples were analyzed using DxFLEX Flow Cytometer (Beckman Coulter, Indianapolis, IN, USA) [81].

### 4.10. Effect of PSO and RA-RF on IL-1β, IL-6, IL-8, TNF-α, and COX-2 mRNA Expression

The effect of PSO and RA-RF on the mRNA expression of pro-inflammatory cytokines such as IL-1β, IL-6, IL-8, TNF-α, and COX-2 in TNF-α-treated A549 cells were analyzed using quantitative reverse transcriptase-polymerase chain reaction (qRT-PCR) [35]. The A549 cells were seeded into a 6-well plate for 24 h, incubated with PSO and RA-RF for 2 h and then stimulated with TNF-α for 24 h. Total RNA was separated using Nucleospin RNA (Macherey-nagel, Germany). Total mRNA was reverse transcribed to cDNA using ReverTra Ace qPCR RT master mix (TOYOBO, Osaka, Japan). The cDNA was subsequently used as the template for qRT-PCR amplification using SensiFAST™ SYBR^®^ Lo-ROX (Bioline Ltd., London, UK). The oligonucleotide primers were received from Bio Basic Inc.; (Toronto, ON, Canada) the primer sequences for pro-inflammatory cytokines are shown as Table 2. The reaction conditions were as follows: 40 cycles of 70 °C for 5 min, 4 °C for 1 min, and 42 °C for 60 min. The target gene expression level was normalized by GAPDH expression level and analyzed using the comparative threshold (ΔΔCt) method (7500 software v2.0.5; Applied Biosystem, Thermo Fisher Scientific Inc., Foster City, CA, USA). Quantitative assays were performed using the following equation: relative quantity (RQ) = 2^−ΔΔCt^ [82]. Then, the values were expressed as mRNA expression relative to the non-treated control.

### 4.11. Western Blot Analysis

The A549 cells were pretreated with PSO and RA-RF for 2 h and co-treated with or without TNF-α. Cells were washed, harvested, and lysed with a lysis buffer containing protease inhibitors and 1% NP-40. Samples were denatured and resolved through sodium dodecyl sulfate-polyacrylamide gel electrophoresis and transferred to nitrocellulose membranes. The membranes were incubated with the appropriate primary antibodies for 24 h and then incubated with secondary antibodies conjugated with horseradish peroxidase for 1 h in 5% non-fat milk. The protein levels were determined using ImageQuant LAS 4000 Mini (GE-Healthcare, Tokyo, Japan) [83].

### 4.12. Statistical Analysis

Statistical analysis was conducted using the one-way ANOVA followed by Dunnett’s test. Data are shown as mean ± standard error of the mean (SEM) value and significant differences were considered at *p* < 0.05, *p* < 0.01, and *p* < 0.001.

## 5. Conclusions

In conclusion, we found that ROS production and p-JNK, FOXO1, and MnSOD expression are significantly increased upon TNF-α stimulation in A549 cells, whereas PSO and RA-RF significantly downregulate those expression. PSO and RA-RF regulate ROS production, resulting in the inhibition of JNK/FOXO1 signaling cascade, which in turn decreases MnSOD expression. Moreover, the molecular mechanism of anti-inflammatory effects of PSO and RA-RF downregulate IL-1β, IL-6, IL-8, TNF-α, and COX-2 via downregulation of JNK phosphorylation and NF-κB protein expression Therefore, PSO and RA-RF from PSM have a potential to be further developed as therapeutic agents for lung inflammation and cancer.

## Figures and Tables

**Table 1:** Percent yield, TPC, TFC and RA content in PSM extract fractions.

Fractions	%Yield	TPC	TFC	RA Content
EtOH	5.63 ± 0.27	84.81 ± 0.85	68.33 ± 1.14	71.72 ± 0.72
HEX	13.53 ± 0.85	4.74 ± 0.80	12.17 ± 1.62	54.56 ± 1.63
DCM	2.76 ± 0.57	198.36 ± 1.67	88.84 ± 4.88	29.42 ± 0.20
EA (RA-RF)	5.33 ± 0.44	248.00 ± 5.14	165.21 ± 2.61	647.68 ± 53.08
Water	23.75 ± 1.34	27.31 ± 0.11	23.56 ± 0.87	37.66 ± 0.19

TPC; total phenolic content (mg GAE/g extract), TFC; total flavonoid content (mg CE/g extract), RA content; (mg/g extract).

**Table 2:** Primers for inflammatory cytokines used in quantitative reverse transcriptase-polymerase chain reaction.

Genes	Forward Sequence	Reverse Sequence
IL-1β	5′-AAA CAG ATG AAG TGC TCC TTC CAG G-3′	5′-TGG AGA ACA CCA CTT GTT GCT CCA-3′
IL-6	5′-ATG AAC TCC TTC TCC ACA AGC-3′	5′-GTT TTC TGC CAG TGC CTC TTT G-3′
IL-8	5′-AGA TAT TGC ACG GGA GAA-3′	5′-GAA ATA AAG GAG AAA CCA-3′
TNF-α	5′-CCC AGG CAG TCA GAT CAT CTT C-3′	5′-AGC TGC CCC TCA GCT TGA-3′
COX-2	5′-CCC TTG GGT GTC AAA GGT AA-3′	5′-GCC CTC GCT TAT GAT CTG TC-3′
GAPDH	5′-GAA GGT GAA GGT CGA GTC A-3′	5′-GCT CCT GGA AGA TGG TGA T-3′

## Data Availability

The data and materials supporting the conclusions of this article are included within the article.
